# Left atrial strain: an operator and software-dependent tool

**DOI:** 10.1186/s13613-024-01331-0

**Published:** 2024-06-21

**Authors:** Christophe Beyls, Osama Abou-Arab, Yazine Mahjoub

**Affiliations:** Department of Anesthesiology and Critical Care Medicine, Amiens University Hospital, 1, rond-point du Pr Cabrol, Amiens, F- 80054 France

Dear Editor,

We read with great interest the article by Cicetti et al. untitled “Effect of volume infusion on left atrial strain in acute circulatory failure” [1]. The authors should be praised for using left atrial strain (LAS), an advanced echocardiographic parameter, for evaluating and predicting fluid responsiveness in patients with acute circulatory failure. In this single-center prospective study, 38 patients were included in the analysis: 45% (*n* = 17/38) in the responder group and 55% (*n* = 21/38) in the non-responder group. LAS values were measured offline using a specific vendor system (EchoPAC, GE Healthcare), employing optimized four-chamber and two-chamber apical views with dedicated automated LAS software. LAS values represent the mean obtained from the four- and two-chamber views before and after administering 500 ml of crystalloid fluid. LAS values were markedly impaired in patients. The authors reported a significant change in all LAS components after volume expansion. None of the LAS components at baseline predicted fluid responsiveness and changes in LAS were not correlated with changes in cardiac ejection.

## In this letter, we would like to outline that several factors may have influenced the study results:

First, the sample size calculation, based on a 5% predicted increase in LASr values after fluid expansion, may be underestimated and may need to account for the variability of LAS measurements related to manual editing, imprecision of apical used views, and repetition of measurements. In this study, the reproducibility of LASr was good (0.88, 95% CI: 0.76–0.94) but not excellent for a dedicated automated LAS mode [2]. This suggests that many patients may have benefited from manual editing of ROIs after automatic left atrium contouring. Moreover, LAS values are derived from the average of LAS values obtained from the two views and over a single cardiac cycle, indicating that manual editing may have been performed twice. Furthermore, manual editing may have been repeated on the measurements taken after fluid expansion, thus repeating measurement errors. Hence, data regarding repeatability and reliability, not only reproducibility, are crucial for echocardiographic studies using a new parameter in critically ill patients [3]. The authors are expected to provide repeatability and reliability of their measurements obtained from each view and over multiple cardiac cycles in the same DICOM loop to determine if the expected 5% variability due to fluid expansion does not solely correspond to the intrinsic variability of the measurement.

Moreover, the authors based the sample size calculation on LAS mean and standard deviation values obtained in previous studies that used manual editing or a different software based only on a 4-chamber view. Comparing measurements obtained with different software and measurement techniques should be done cautiously because significant variability in LAS measurements depending on the software has been previously reported [4].

Second, in the group of patients labeled as non-responders, all hemodynamic parameters (heart rate, mean arterial pressure, systolic, and diastolic pressure) significantly improved after fluid expansion without changing their LVOT-VTI. Surprisingly, this is the clinically expected and desired outcome after volume expansion, especially if vasoactive drugs remain unchanged. As for many studies assessing fluid responsiveness, the evaluation technique to diagnose the patient as a responder is crucial. In their study, the authors defined responders as patients with an increase of 10% or more in LVOT-VTI after volume expansion. This threshold may have led to misclassification. In critical care, the precision of LVOT-VTI varies from 4 to 14% depending on the observers and the presence of mechanical ventilation [5].

Third, the study population is highly heterogeneous and includes patients who may have severe pre-existing left atrial dysfunction due to their medical history. The authors did not compare demographic data between the two groups. Even though the baseline LASr value is comparable and severely impaired in both groups, acute and chronic impairments may respond differently to an acute change in load or perfusion pressure.

To conclude, further evaluation of new and promising echocardiographic parameters, such as LASr, is necessary in ICU settings to prevent premature dismissal.

## Data Availability

Not applicable.
